# 

**Published:** 1885-10

**Authors:** 


### CONTENTS:

Art. I—Nervous Energy, By Dr. E. Parsons....241
Art. II—Pulpless Teeth, By Dr. Wilson....253
Art. III—Dead Teeth in the Jaws, By Truman W. Brophy, M. D., D. D. S....260
Art. IV—Diagnosis and Treatment of Dentritic Cystic Tumors of the Jaws, By John S. Smith, D. D. S....265
Art. V—Thoroughness, By L. P. Dotterer, D. D. S....269
Art. VI—What Fillings Should we Use, By Dr. W. G. A. Bonwill....272
Art. VII—Some Methods of Separating Teeth with Wedges, By Dwight M. Clapp..274
Art. VIII—Cocaine, By W. W. Allport, M. D., D. D. S....278

### EDITORIAL, ETC.

University of Maryland Dental Department....280
Correspondence....281

### BIBLIOGRAPHICAL.

A Series of Questions Pertaining to the Curriculum of the Dental Student, By Ferdinand J. S. Gorgas, A. M., M. D., D. D. S., of the University of Maryland....283
Practical and Analytical Chemistry, By Henry Trimble, Ph. G....283
Chemical Problems, By Dr. Karl Stammer....284
Quiz Questions, By Prof. J. F. Flagg, M. D., D. D. S....284

### MONTHLY SUMMARY.

Peroxide of Hydrogen....285

Alcoholic Paralysis....286

Nourishing the Tissues of the Teeth....288

